# Supplementary material for: Aldehyde-alcohol dehydrogenase undergoes structural transition to form extended spirosomes for substrate channeling
Source: Commun Biol. 2020 Jun 10;3:298. doi: 10.1038/s42003-020-1030-1 (PMC7286902; doi:10.1038/s42003-020-1030-1)
Supplement: Supplementary file 1 — Supplementary Information [file 42003_2020_1030_MOESM1_ESM.pdf]

Supplementary Figure 1

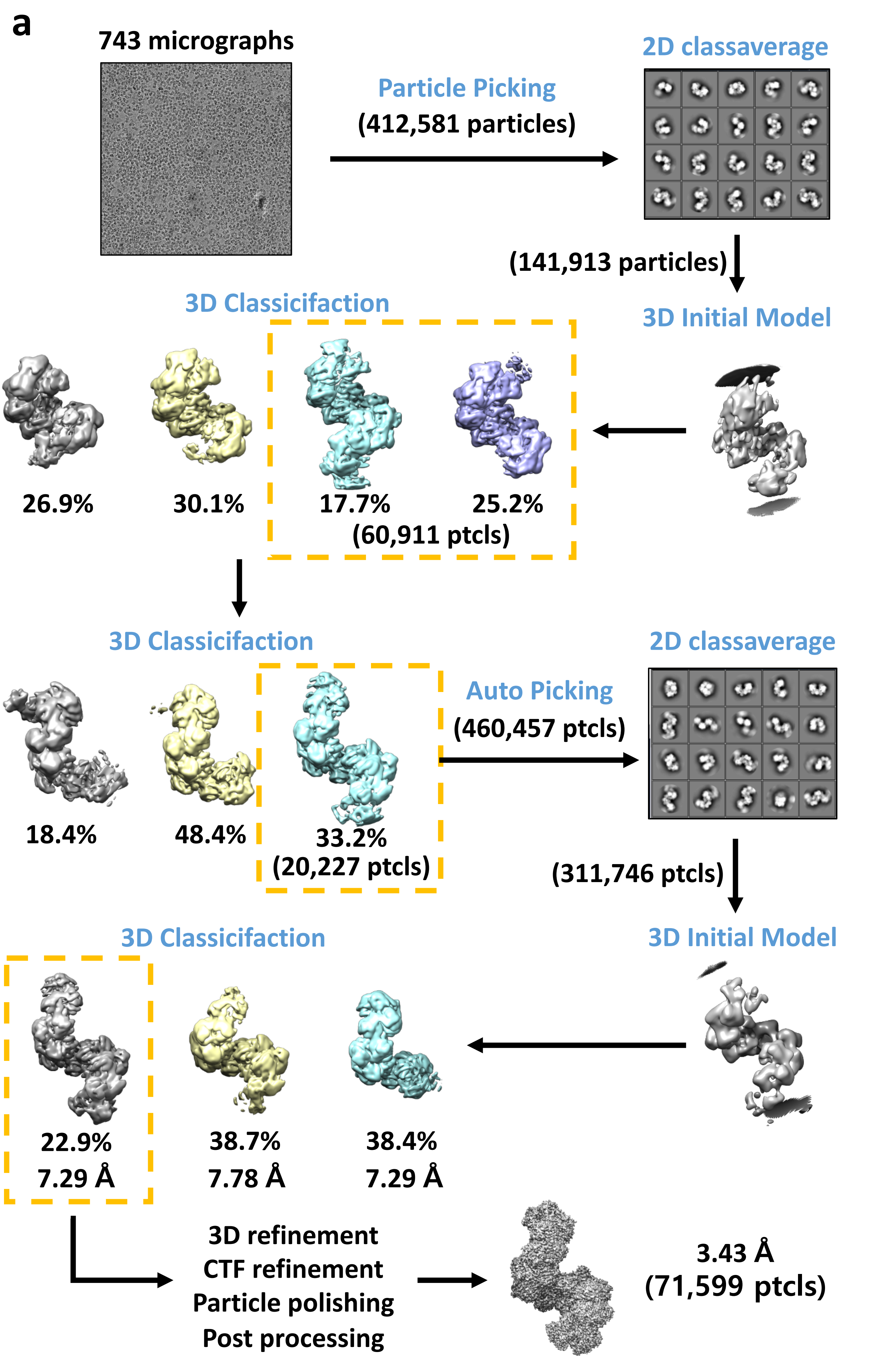

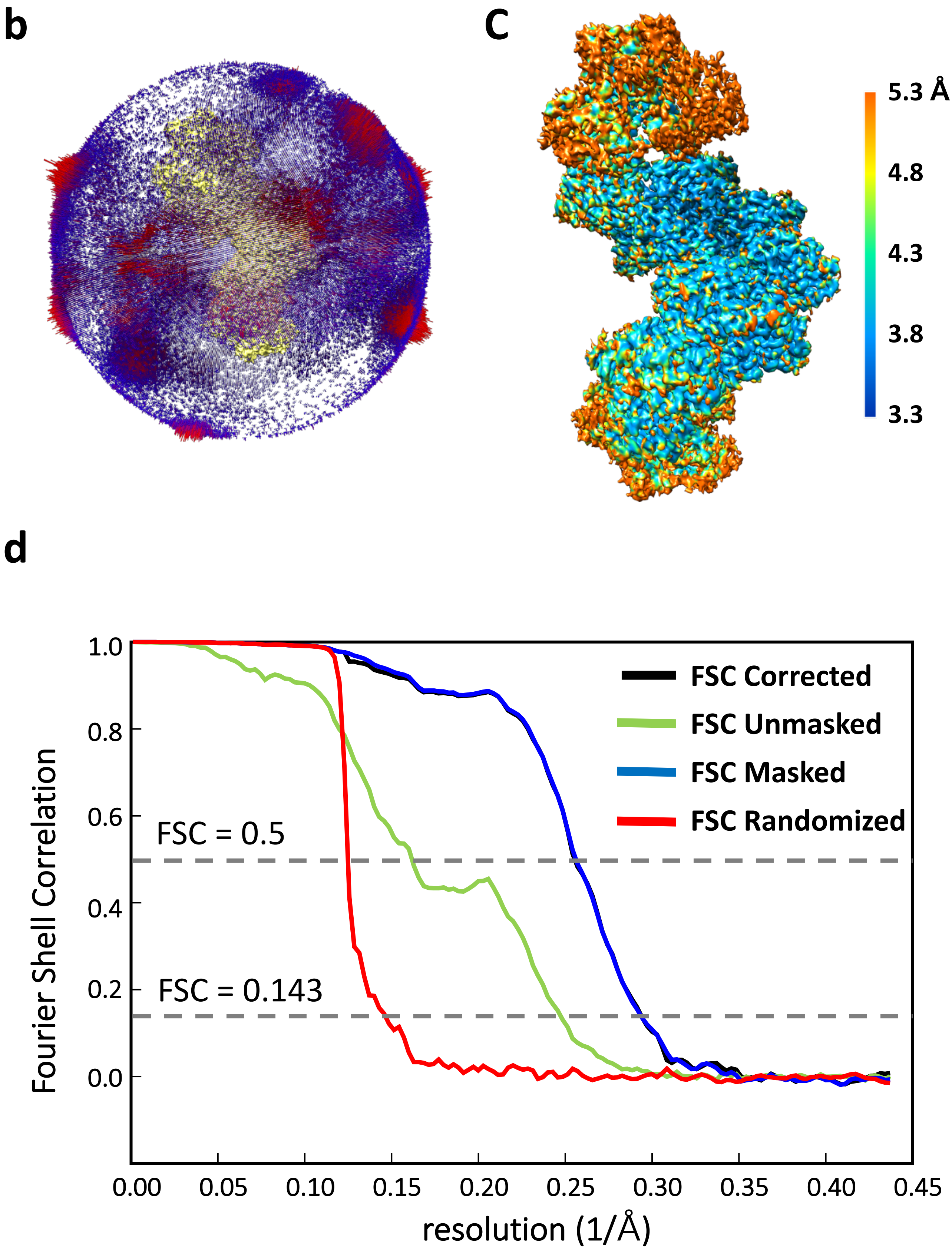

Supplementary Figure 1. Cryo-EM imaging process

a. A flow chart for the cryo-EM image processing. **b**. Euler angle distribution of the particles used for the final cryo-EM structure. **c**. A local resolution heat map of AdhE on the cryo-EM map. The local resolution was estimated using Relion3.0[19]. The color code indicates local resolutions. **d**. FSC curves calculated show the resolution of 3.43 Å and 3.90 Å by an FSC coefficient criterion of 0.143 and 0.5 respectively.

Supplementary Figure 2

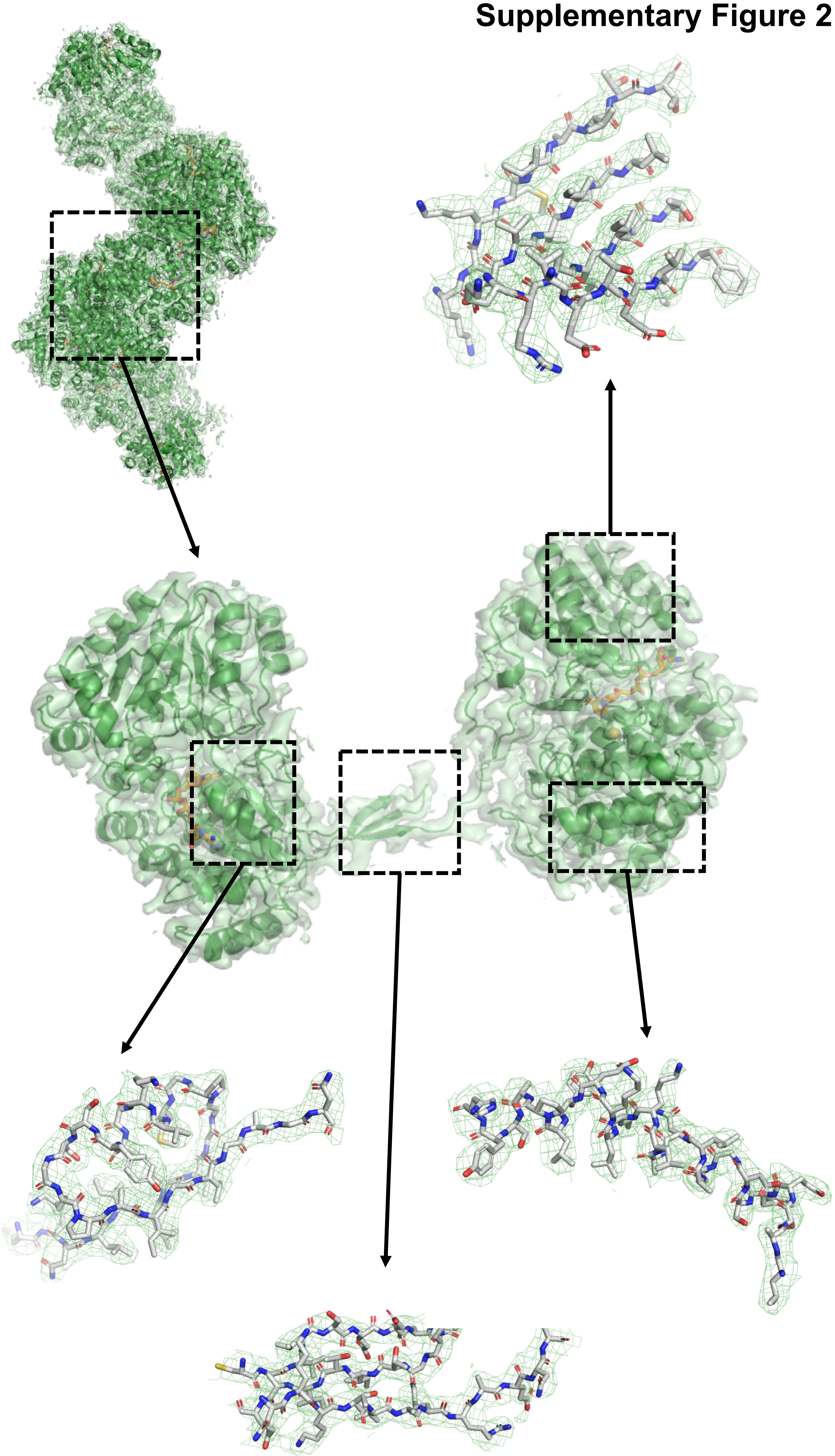

Supplementary Figure 2. Cryo-EM map of AdhE

Atomic models were fitted in the cryo-EM map at several regions of AdhE.

Supplementary Figure 3

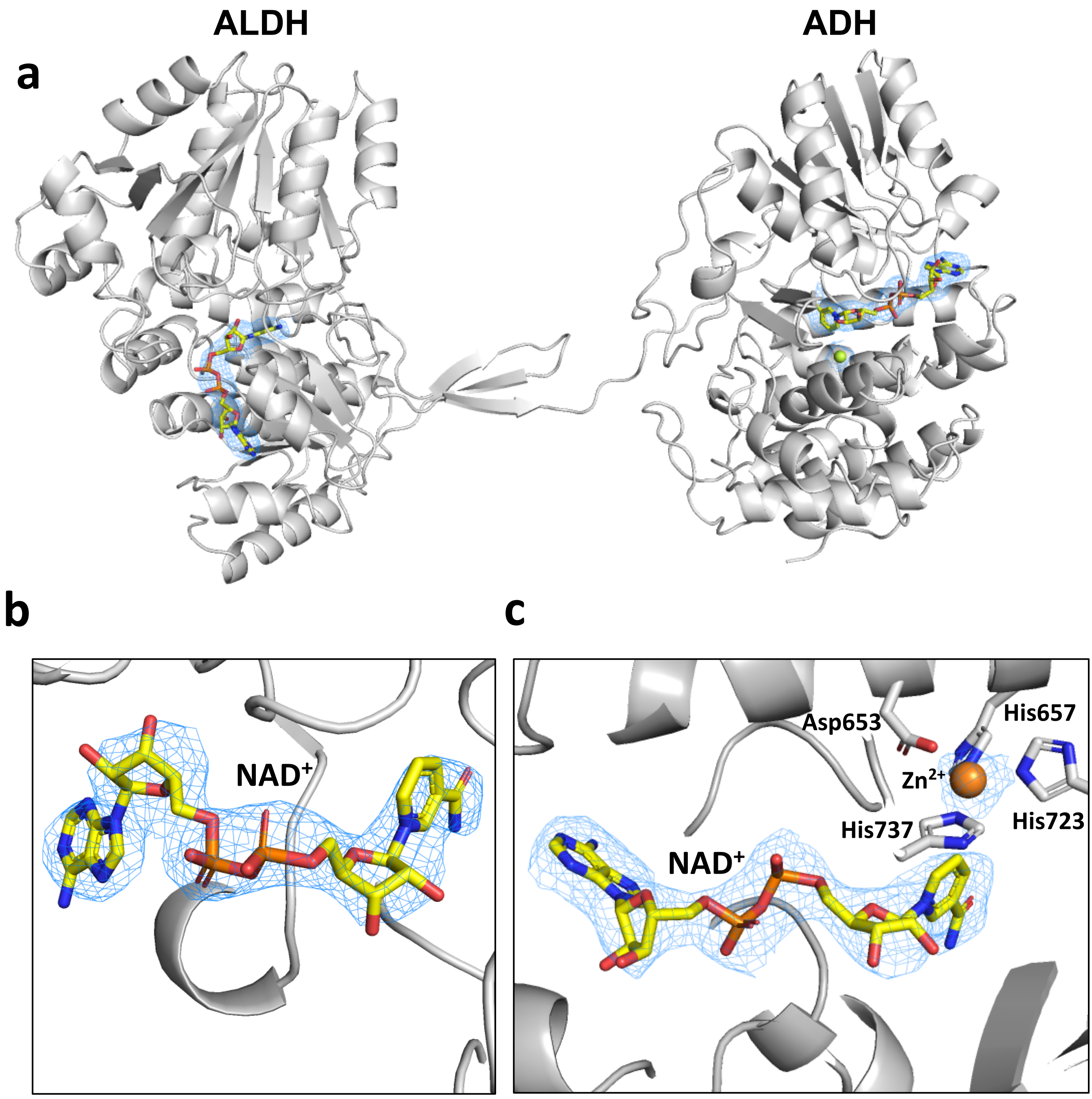

Supplementary Figure 3. The binding of NAD+ and Zn2+ at the active site in the ALDH and ADH domains

a. The atomic model with cryo-EM maps near NAD^{+} shows the location of the cofactor binding site. **b-c.** Cryo-EM maps near NAD^{+} shows the configurations of NAD^{+}s in the ALDH (b) and ADH (c) domains. Zn^{2+} in the ADH domain is coordinated by three histidines (His657, His723 and His737) and asparate (Asp653) (c).

Supplementary Figure 4

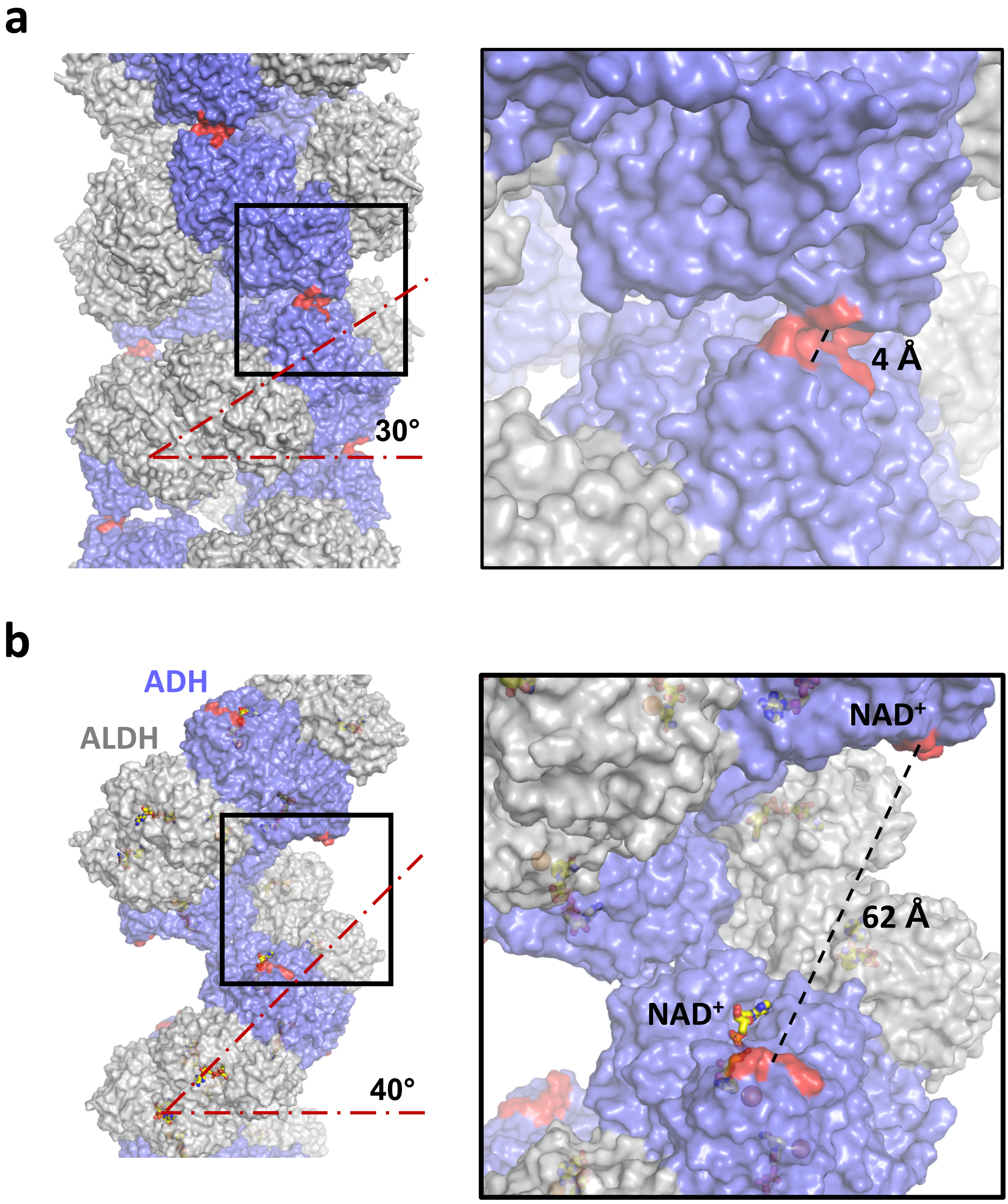

Supplementary Figure 4. The cryo-EM structures of AdhE in compact and extended forms

The cryo-EM structures of AdhE in a compact form (a) and an extended form (b) in a surface presentation in the same view as Fig. 3. The ADH catalytic site is shown in red. In the compact form, The neighboring ADH catalytic sites are separated by only 4 Å, whereas this increases to 62 Å in the extended spirosome. The angle of the helical turn relative to the helical axis is 30° in the compact spirosome, while 40° in the extended form.

Supplementary Fig. 5

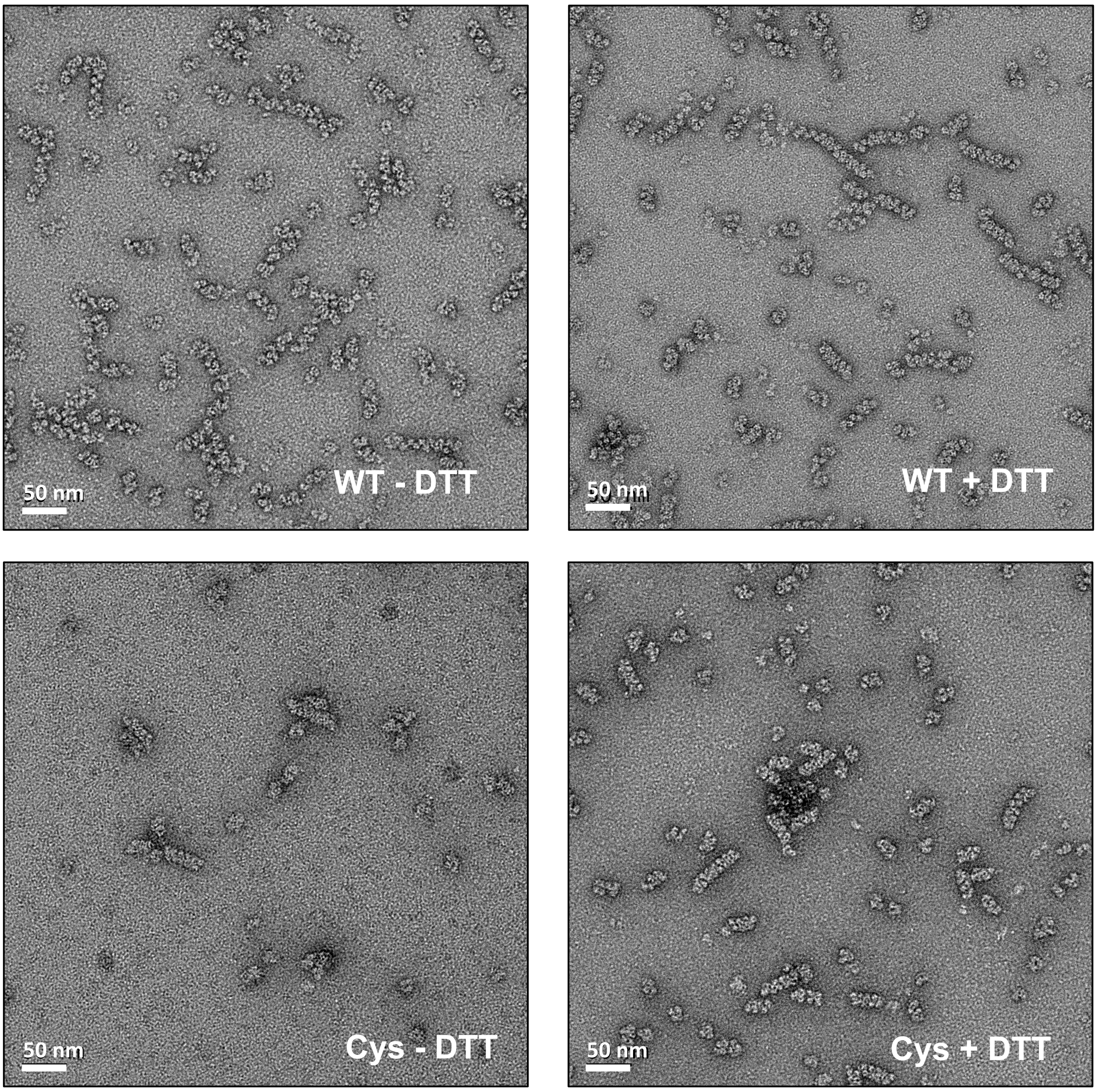

Supplementary Fig. 5. Cryo-EM analysis of WT and Cys mutant AdhE spirosomes in the presence and absence of reducing agent

Negative stained EM analysis of the wild-type and Cys mutant AdhE spirosomes in the absence and presence of DTT.

Supplementary Figure 6

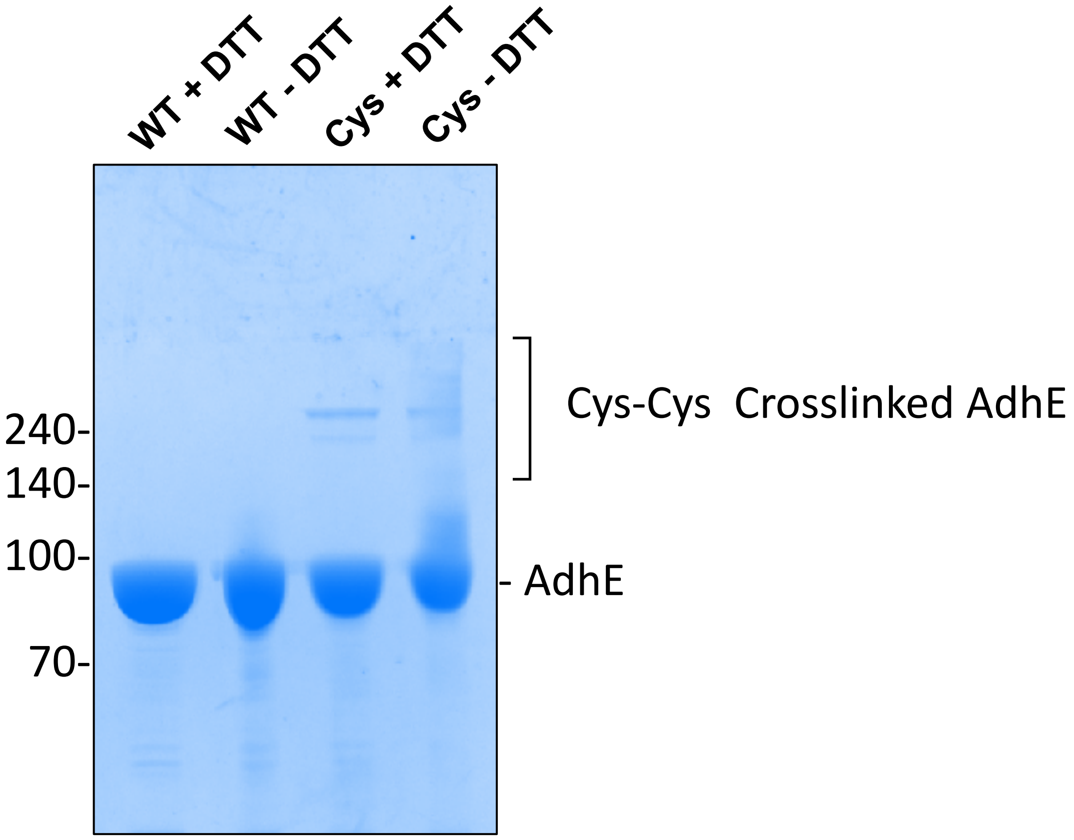

Supplementary Figure 6. SDS-PAGE analysis of Cys-Cys crossled AdhE
The crosslinked AdhE mutants in the absence (-DTT) and presence (+DTT) of DTT.

Supplementary Table 1. Refinement statistics

	AdhE Extended Spirosome (Zn, NAD, and EtOH)
**Sample Preparation**	
Grid	Quantifoil R2/2 200 mesh
Cryo-specimen freezing	Vitrobot IV
**Data Collection**	
Electron Microscope	Talos Glacios (200 keV)
Detecting device	Falcon III (Electron counting mode)
Total electron exposure/used (e/Å^2)	40.76 / 40.76
Defocus range (μm)	-0.8 ~ -2.8
Pixel size (Å^2)	1.14
**Processing program**	Relion
Obtained / Used micrographs (no.)	1,704 / 743
Initial / Final particles used (no.)	412,581 / 71,599
Symmetry imposed	C1
FSC threshold	0.143
**Resolution (Å)**	3.43
**Refinement program**	PHENIX
**Model composition**	
Nonhydrogen atoms	40,146
Protein residues	5,214
Zn^{2+} / NAD^+	6 / 12
**R.m.s. Deviation**	
Bond Length (Å)	0.010
Bond Angle (°)	0.688
**Validation**	
MolProbity Score	1.99
Clash Score	5.33
**Ramachandran Plot**	
Favored / Allowed (%)	95.66 / 3.34
Ourliers (%)	0.00
**Mask CC**	0.79
