# Supplementary material for: Aldehyde-alcohol dehydrogenase undergoes structural transition to form extended spirosomes for substrate channeling
Source: Commun Biol. 2020 Jun 10;3:298. doi: 10.1038/s42003-020-1030-1 (PMC7286902; doi:10.1038/s42003-020-1030-1)
Supplement: Supplementary file 6 — Description of Additional Supplementary Files [file 42003_2020_1030_MOESM6_ESM.pdf]

Descriptions of Additional Supplementary Files

Supplementary Movie 1: Structural transition of the AdhE compact and extended forms

The morphing two structural states of AdhE dimer from the compact to extended forms. Each monomer is colored in cyan and light blue colors.

Supplementary Movie 2: Structural transition of the AdhE compact and extended spirosome

The morphing two structural states of AdhE spirosome from the compact to extended forms in a dimer. Each monomer is colored in cyan and light blue colors.

Supplementary Movie 3: Docking simulation of Acetaldehyde in the substrate channel between ALDH and ADH domain

The docking simulation is an ensemble simulation which evolves the positions of an ensemble of molecules with iteration. In this movie, only 100 lowest-energy positions of 600 positions are shown. Molecules may move abruptly in this simulation because the move sets are stochastic, involving cross-overs and mutations in the molecular conformational space. This is different from a conventional molecular dynamics simulation which computes a continuous time trajectory of molecular motions. Molecular occupation along the channel is pretty high even at the early stage of simulation, implying that the channel could be easily recognized by short local energy optimizations performed at the beginning of the simulation.

Supplementary Data: Raw data for the enzymatic activity assay shown in Fig. 5C
